# Sequencing and Analysis of the Entire Genome of the Mycoparasitic Fungus Trichoderma afroharzianum

**DOI:** 10.1128/MRA.00211-21

**Published:** 2021-04-15

**Authors:** Anastasia Landeis, Markus Schmidt-Heydt

**Affiliations:** a Max Rubner-Institut, Federal Research Institute of Nutrition and Food, Department of Safety and Quality of Fruit and Vegetables, Karlsruhe, Germany; Vanderbilt University

## Abstract

*Trichoderma* sp. is a globally occurring fungal ascomycete. The genus *Trichoderma* belongs to the order of Hypocreales in the class of Sordariomycetes. Due to its importance as a mycoparasite and biocontrol fungus that antagonizes phytopathogenic and mycotoxin-producing fungi, the genome of the Trichoderma afroharzianum strain BFE349 from the fungal strain collection of the Max Rubner-Institut was sequenced and analyzed.

## Abstract (precis)

*Trichoderma* sp. is a globally occurring fungal ascomycete. The genus *Trichoderma* belongs to the order of Hypocreales in the class of Sordariomycetes. Due to its importance as a mycoparasite and biocontrol fungus that antagonizes phytopathogenic and mycotoxin-producing fungi, the genome of the Trichoderma afroharzianum isolate BFE349 from the fungal strain collection of the Max Rubner-Institut was sequenced and analyzed.

## ANNOUNCEMENT

*Trichoderma* spp. colonize plant roots, and some species are rhizosphere competent so they grow directly on roots and support the vitality and root growth of plants while inducing their systemic resistance to phytopathogens (1, 2). As an important mycoparasite and biocontrol fungus, Trichoderma afroharzianum feeds on fungal material and actively attacks and parasitizes other fungi (3). It is known that *Trichoderma* spp. combat most pathogenic fungi, such as *Fusarium* and *Alternaria* members, but also saprophytic species, such as those from *Penicillium* and *Aspergillus* (4, 5). However, *Trichoderma* spp. are also a major source of contamination and crop loss in mushroom farms (6, 7). *Trichoderma afroharzianum* BFE349 was isolated from field soil in South Germany, which is a common habitat for this species of fungi. For isolation of genomic DNA, the strain was grown 7 days at 25°C in yeast extract-saccharose (YES) broth (20 g/liter yeast extract and 150 g/liter saccharose). Genomic DNA of *T. afroharzianum* BFE349 was extracted from a pure culture using the NucleoSpin plant II kit (Macherey-Nagel) and quantified and quality checked using a NanoDrop 1000 instrument (VWR International) and Qubit 3.0 photometer, respectively. Genome sequencing was carried out on the MiSeq platform (Illumina) as follows: the sequencing library was built using the Illumina Nextera DNA XT kit and quality checked using Experion DNA 1k analysis (Bio-Rad Laboratories). Raw reads (read length of 2 × 300 bp) were processed with the FASTQ preprocessing toolkit (Blast2Go Pro V5.2). *De novo* assembly was carried out with SeqMan NGen V17.2 (Lasergene). Default parameters were used except where otherwise noted; sequencing adapters, PhiX control, contigs of <200 nucleotides (nt), and mitochondrial sequences were removed. The assembly size was 38,392,485 bp with 60× coverage which contained 962 genomic scaffolds/contigs; the *N*_50_ value was 77,771 kb, the *L*_50_ value was 147 kb, and the G+C content was 49.6%. The prediction of biosynthetic gene clusters (BGCs) was carried out with antiSMASH fungal V6.0 alpha using the cluster finder algorithm for BGC border prediction with standard settings (8, 9).

Within the genome sequence of *T. afroharzianum* BFE349, a total of 56 BGCs were predicted, as follows: 16 T1PKS clusters, 18 NRPS clusters, 7 PKS-NRPS hybrid clusters, 1 fungal-RIPP cluster, 5 saccharide clusters, 2 fatty acid clusters, and 7 terpene clusters. The predicted BGCs show similarity to known BGCs; one predicted BGC shows a gene sequence similarity (gss) of 33% to the depudecin gene cluster. Depudecin is a deacetylase inhibitor and virulence factor described in Alternaria brassicicola (10). In addition, one predicted BGC shows a gss of 75% to the gene cluster for tricholignan A biosynthesis. Tricholignan is used by the producing fungus to facilitate reductive iron assimilation in plants (11). Other predicted BGCs show gene sequence similarities to chaetoviridin, which is an azaphilone antibiotic (gss of 27%) (12); the cytotoxic beta-lactone aspyridone A (gss of 33%) (13); curvupallide (gss of 22%) (14); the pigment naphthopyrone (gss of 100%) (15); squalestin S1 (gss of 40%) (16); the xanthone heterodimer neosartorin (gss of 15%) (17); the siderophore dimethylcoprogen (gss of 100%) (18); the tetracyclic diterpene fungal antibiotic sordarin (gss of 26%) (19), and last but not least the terpene copalyl diphosphate (gss of 28%) (20). Future analyses of the above-mentioned interesting secondary metabolites and molecular characterization of the respective gene clusters expressed in *Trichoderma* spp. will allow conclusions to be drawn about their importance for the mycoparasitic state, for the ability to complex and assimilate iron, and to support plants in their growth.

### Data availability.

This whole-genome shotgun project has been deposited in NCBI/SRA GenBank under accession no. JAEKOX000000000.1 and PRJNA682927.
